# Develop a preliminary core germplasm with the novel polymorphism EST-SSRs derived from three transcriptomes of colored calla lily (*Zantedeschia hybrida*)

**DOI:** 10.3389/fpls.2023.1055881

**Published:** 2023-02-02

**Authors:** Yi Wang, Tuo Yang, Xue Wang, Xuan Sun, Hongyan Liu, Di Wang, Huanxiao Wang, Guojun Zhang, Yanbing Li, Xian Wang, Zunzheng Wei

**Affiliations:** ^1^ Institute of Grassland, Flowers and Ecology, Beijing Academy of Agriculture and Forestry Sciences, Beijing, China; ^2^ College of Horticulture, China Agricultural University, Beijing, China; ^3^ Hebei Key Laboratory of Horticultural Germplasm Excavation and Innovative Utilization, College of Horticultural Science & Technology, Hebei Normal University of Science & Technology, Qinhuangdao, China; ^4^ Landscape Engineering Technology Research Center, Zhoukou Normal University, Zhoukou, China

**Keywords:** colored calla lily, transcriptome, EST-SSRs, core germplasm, genetic diversity

## Abstract

The development of high-throughput sequencing technology has made it possible to develop molecular markers such as EST-SSR from transcriptome sequences in non-model plants such as bulbous flowers. However, the EST-SSR markers that have been developed are weakly validated and low polymorphic due to the short read size and poor quality of the assembled sequences. This study therefore used the CandiSSR pipeline to identify 550 potential polymorphic SSR loci among 487 homologous unigenes based on the transcriptomic sequences of three varieties of colored calla lily, and 460 of these loci with appropriate flanking sequences were suitable for primer pairs design. A further validation with 200 randomly selected EST-SSRs demonstrated an increase of more than 30% and 100% in amplification validity and polymorphism, respectively, in comparison with our previous study. In addition, since most of the current varieties of colored calla lily are hybridized from a few species, which have low genetic diversity, we subsequently identified primary core germplasm for 160 colored calla lily accessions using the aforementioned 40 polymorphic EST-SSRs. It was concluded that the core germplasm containing 42 accessions derived from the M strategy incorporated into the software Power Core was the most representative of all 160 original germplasm, as evidenced by the preservation of 100% of the EST-SSR variation, with a higher level of genetic diversity and heterogeneity (*Nei* = 0.40, *I* = 0.66, *PIC* = 0.43). This study provides a practical example of polymorphism EST-SSR markers developed from multiple transcriptomes for non-model plants. A future breeding program for colored calla lily will also benefit from the core germplasm defined by those molecular markers.

## 1 Introduction

The calla lily, a perennial bulbous flowering plant referring to the *Zantedeschia* genus of the Araceae family, is indigenous to the wetlands, grassy slopes, and forest boundaries of southern Africa (Singh et al., 1996). Colored calla lily, also known as *Zantedeschia hybrida*, are mainly inter- or intra-species hybrids of *Z. albomaculata*, *Z. elliottiana*, *Z. pentlandii*, and *Z. rehmannii*, with flowers varying from deep red to pink, orange to yellow, and even white. They are well-known around the worldwide due to their exceptionally high decorative value and extended flowering duration. Hundreds of varieties or hybrids are now available as a profitable international crop in New Zealand, the Netherlands, and the United States. Previous research has shown that molecular marker is useful genetic tool in crop marker-assisted selection breeding. With the exception of a report that used Random Amplified Polymorphic DNA (RAPD) primarily for cultivar identification and genetic diversity (Hamada and Hagimori, 1996), such an approach has been utilized very infrequently in the exploration of colored calla lily. It is probable that the dominant pattern of inheritance for this type of marker renders identification of allelic diversity challenging.

Simple Sequence Repeat (SSR) markers are being emerging recently because they are multi-allelic, prevalent all over the genome, repeatable, polymorphic, and can be genotyped quickly (Morgante et al., 2002). Among which, Expressed Sequence Tag-SSRs (EST-SSRs) derived from transcribed DNA regions, the majority of which are situated in functional genes, are anticipated to be the most transferrable across species, making them ideal for linkage map construction and comparative mapping. EST-SSR has been developed for a variety of bulbous crops (Wang et al., 2018; Hiremath et al., 2021), including colored calla lily, as a result of our prior research. In *Z. rehmannii*, 200 pairs of primers designed from transcriptome-assembled unigenes were randomly examined, revealing that 137 pairs had amplification products and 58 pairs possessed polymorphisms (Wei et al., 2016). While we recognize the benefits behind using EST-SSR markers in genetic breeding, we also highlighted that the conventional approach to development EST-SSR markers has high costs and poor polymorphic, which may limit their future usage (Morgante et al., 2002; Thiel et al., 2003). To address or improve the limited number of polymorphic EST-SSR markers eventually developed from a single transcriptome-derived unigenes, Xia et al. (2016) recently established a methodology known Candidate polymorphic SSR (CandiSSR), a bioinformatics algorithm based on multiple transcriptome or genome sequences, it was written to execute using the Perl language. Further experiments confirmed that the randomly selected polymorphic EST-SSR primers amplified with 100% efficiency and achieved more than 90% polymorphism, which is higher than those developed from a single transcriptome-derived unigenes alone and provides a tool to develop polymorphic EST-SSRs for crops like colored calla lily with no reference genome.

The construction and administration of a plant collection comprising many individuals is essential for the selection and development of innovative crop varieties. However, it requires extensive space, skilled staff, and plant identification skills, which may result in a more expensive process. Given the high degree of redundancy and similarity in those initial crop collections, it is vital to establish a core collection of unique and distinctive germplasm materials (Zhang et al., 2011). Typically, the proportion of core germplasm in locally and globally cultivated plants ranges from 5% to 40% of the original germplasm (Wang et al., 2007; Escribano et al., 2008; Liang et al., 2014; Wang et al., 2014). Molecular markers have been shown to be favorable for defining the core germplasm due to their capacity to reflect variation at the genome level (Santesteban et al., 2009). Combined with the Maximisation (M) strategy, which attempts to maintain the maximum number of alleles present at each locus (Brown and Schoen, 1994), the core collections have been developed using SSRs for many plant species, such as grape, olive, sesame, cassava, capsicum, and cashaw (Le Cunff et al., 2008; Belaj et al., 2011; Zhang et al., 2012; Lee et al., 2016; Mohana and Nayak, 2018). Given that each entry in the core collection is identified based on the allelic potential of its molecular maker, regardless of its phenotypic variability, this will effectively cut down on wasted work and make the crop breeding program more efficient.

It is also essential to identify the colored calla lily’s core germplasm using molecular markers such as SSR or EST-SSR. Due to the narrow range and plastome-genome incompatibility among wild species (Yao et al., 1994), the majority of existing varieties of colored calla lily are the choice of ongoing crossings between several specie individuals, with minimal variation and low genetic diversity across varieties (Wei et al., 2017). This makes it challenging to generate new colored calla lily varieties *via* conventional hybrid breeding. Using the CandiSSR pipeline, we firstly identified distinct conserved polymorphic EST-SSR markers based on three transcriptome assembled sequences of colored calla lily. Then we performed a genetic diversity analysis of 160 accessions based on the markers information and finally established a representative core germplasm using the M strategy. This research offers us a beneficial molecular marker-assisted tool that will enhance breeding efficiency and accelerate the development of new varieties of colored calla lily in the future.

## 2 Materials and methods

### 2.1 Plant materials and DNA/RNA extraction

A primary germplasm collection of colored calla lily was maintained at the Bulb and Perennial Flowers Genebank Collection, Yanqing Farm, Beijing Academy of Agriculture and Forestry Sciences (Beijing, China) for the purpose of this study. These are 160 accessions in this collection, the majority of which originate from New Zealand, the Netherlands, the United States, and China. All of these accessions are detailed in **Supplementary Table S1**. The young leaves of each accession were collected individually for DNA extraction, while spathes and bulbs of *Z. hybrida* cv. Florex Gold and Black Magic were used for RNA extraction, respectively. Each sample’s genomic DNA and total RNA were respectively extracted using the DNeasy Plant Mini Kit and RNeasy Plant Mini Kit (Zexing Biotech, Beijing, China). The quantity of genomic DNA or total RNA was checked by resolution in the 2.4% (w/v) agarose gel and determined with the NanoDrop™ One.

### 2.2 Transcriptome sequencing, *de novo* assembly and annotation

Following synthesis of cDNA with the Thermo Frist Strand cDNA Synthesis Kit (Thermo Fisher Scientific), ten cDNA libraries were generated, nine for Florex Gold’s spathes and one for Black Magic’s bulb, which contained approximately 130-150 bp insertion fragments by using Illumina TruSeq RNA Sample Prep Kits (Illumina, Santiago), and then subjected to paired-end sequencing on an Illumina HiSeq 2000 or 2500 platform. The raw data for Florex Gold and Black Magic were deposited to the Genome Sequence Archive. Moreover, our previously published transcriptome of variety Rehmannii (Wei et al., 2016), which specialized in mixed tissues (spathes, leaves, bulbs, stems, etc.), was also retrieved from the National Center for Biotechnology Information for further analysis.

Since transcriptome sequencing has been conducted on different platforms, a unified data processing platform incorporated into the website (http://www.biocloud.net/, Beijing Biomarker Technologies Co., Ltd) has been first used to evaluate raw reads of those three varieties. After eliminating the sequence adapters and low-quality reads, the clean reads were *de novo* assembled, respectively, using Trinity software (Grabherr et al., 2011) with default parameters. The longest single gene-transcript was then extracted to generate the unigenes library for each variety, which was compared with multiple protein databases using BLAST (Altschul et al., 1997) and HMMER (Finn et al., 2014) with a significant E-value cut-off (10E-5 or 10E-10). The databases included the Non-Redundant Protein Sequence Database (NR), the Swiss-Prot Protein Sequence Database (Swiss-Prot), the Cluster of Orthologous Genes (COG), the Gene Ontology (GO), and the Kyoto Encyclopedia of Genes and Genomes (KEGG).

### 2.3 Identification of EST-SSR in transcriptome-derived unigenes

As previously described by; (Wei et al., 2012; 2016), the MIcroSAtellite identification tool (MISA, http://pgrc.ipk-gatersleben.de/misa/misa.html) was initially used to identify microsatellites for each variety’s assembled unigenes. The minimum number of repeats used to select the SSRs was ten for mononucleotide repeats, six for dinucleotide repeats, and five for tri-, tetra-, penta-, and hexanucleotide repeats. Furthermore, CandiSSR (Xia et al., 2016), a pipeline that runs in a single Perl script, was further used to identify candidate polymorphic SSR across three transcriptomes of colored calla lily. This methodology could be summarized as follows: (i) retrieve SSRs (also including mononucleotide repeats) in the reference transcriptome of Florex Gold; (ii) align the transcriptome sequences of the other two varieties to the flanking sequences of the previously identified reference SSRs with BLAST; (iii) extract the non-reference sequences of valid hits after removing low-quality results using the criterion of Minimum Identity and Minimum Coverage; (iiii) re-identify and generate a final list that includes specific referenced SSRs; and (v) filter out polymorphic SSRs with Standard Deviation = 0 and Miss Rate > 50%. Finally, after removing unigenes with verified SSR makers, as reported by Wei et al. (2016), primer pairs were designed using Primer Premier 5.0 for the remaining unigenes with potential novel polymorphic SSRs. In addition, those filtered unigenes used for SSR design were reapplied to align multiple protein databases using the BLASTX program, as described before.

### 2.4 Novel polymorphic EST-SSR marker amplification and evaluation

To screen EST-SSR primer pairs designed in this study, 21 accessions previously applied in Wei et al. (2016) were initially used. The PCR amplification of each EST-SSR marker is performed at its optimal annealing temperature, according to the protocol described by; (Wei et al., 2012; 2016). Silver staining was used to identify PCR products separated on 8.0% polyacrylamide gel (Wang et al., 2018; Xiong et al., 2020; Xiong et al., 2021). The product sizes were estimated for each marker by comparison with a 100-300 bp DNA marker. A further genetic diversity analysis of 160 colored calla lily accessions was conducted using the polymorphic EST-SSR markers screened above. The observed number of alleles (*Na*), the effective number of alleles (*Ne*), observed heterozygosity (*Ho*), expected heterozygosity (*He*), Nei’s expected heterozygosity (*Nei*), as well as the Shannon information index (*I*) and polymorphic information content (*PIC*) were respectively calculated using GenAlEx 6.4 (Peakall and Smous, 2010), Popgene (Yeh et al., 1997) and Power Marker Version 3.25 (Liu and Muse, 2005).

### 2.5 Define the core germplasm

Three distinct strategies were used to determine the core germplasm of colored calla lily employing 40 EST-SSR allelic information from 160 accessions. The M strategy applied in the program Power Core (Kim et al., 2007) primarily maximizes the number of alleles per sample by iteratively removing redundant germplasm Power Core supports the development of the core set by reducing redundancy of useful alleles, thereby increasing its richness. First, we pasted the data into the software - power core, and click classifying, random run, finally, the core germplasm is obtained. Using the core set function, the simulated annealing (SA) approach in the Power Marker 3.25 program increases allelic richness for each sampling (Garcia-Lor et al., 2017). Both SANA-PM(SA) and SAGD-PM(SD) methods use power marker software and are annealing strategies, but SA is based on alelele number and SD is based on gene diversity. MR (Modified Rogers distance) and SH (Shannon’s Diversity Index) for different sample ratios and weights were the most crucial elements incorporated in Core Hunter software on Linux environments (Thachuk et al., 2009). GraphPad Prism 8.4.3 (GraphPad Software Inc., USA: http://www.graphpad.com/) was used to statistical analysis of *P* value. *, *P* < 0.05; **, *P* < 0.01; ***, *P* < 0.001; ****, *P* < 0.0001.

## 3 Results

### 3.1 *De novo* assembly and annotation of transcriptome sequences of three varieties

All raw reads were processed by sequential quality control to generate clean, high-quality reads, and then *de novo* assembly was performed using the Trinity software. The three varieties of Florex Gold, Rehmannii, and Black Magic were reassembled to yield 109,286, 89,825, and 120,836 unigenes, respectively. The N50 length of each variety was 1030 bp, 960 bp, and 825 bp, while their respective average lengths were 611.7 bp, 604.4 bp, and 585.7 bp (**Supplementary Table S2**). As indicated in **Table S2**, for each variety, the number of unigenes continuously decreased as the length of the unigene increased. In Florex Gold, Rehmannii, and Black Magic, 51,121 (46.78%), 39,715 (44.21%), and 50,586 (41.86%), respectively, of unigenes with a length between 200 bp and 300 bp were found, whereas the number of unigenes with lengths greater than 2000 bp was the lowest at 6,466 (5.92%), 4,958 (5.52%), and 5,944 (4.92%).

**Figure 1:**
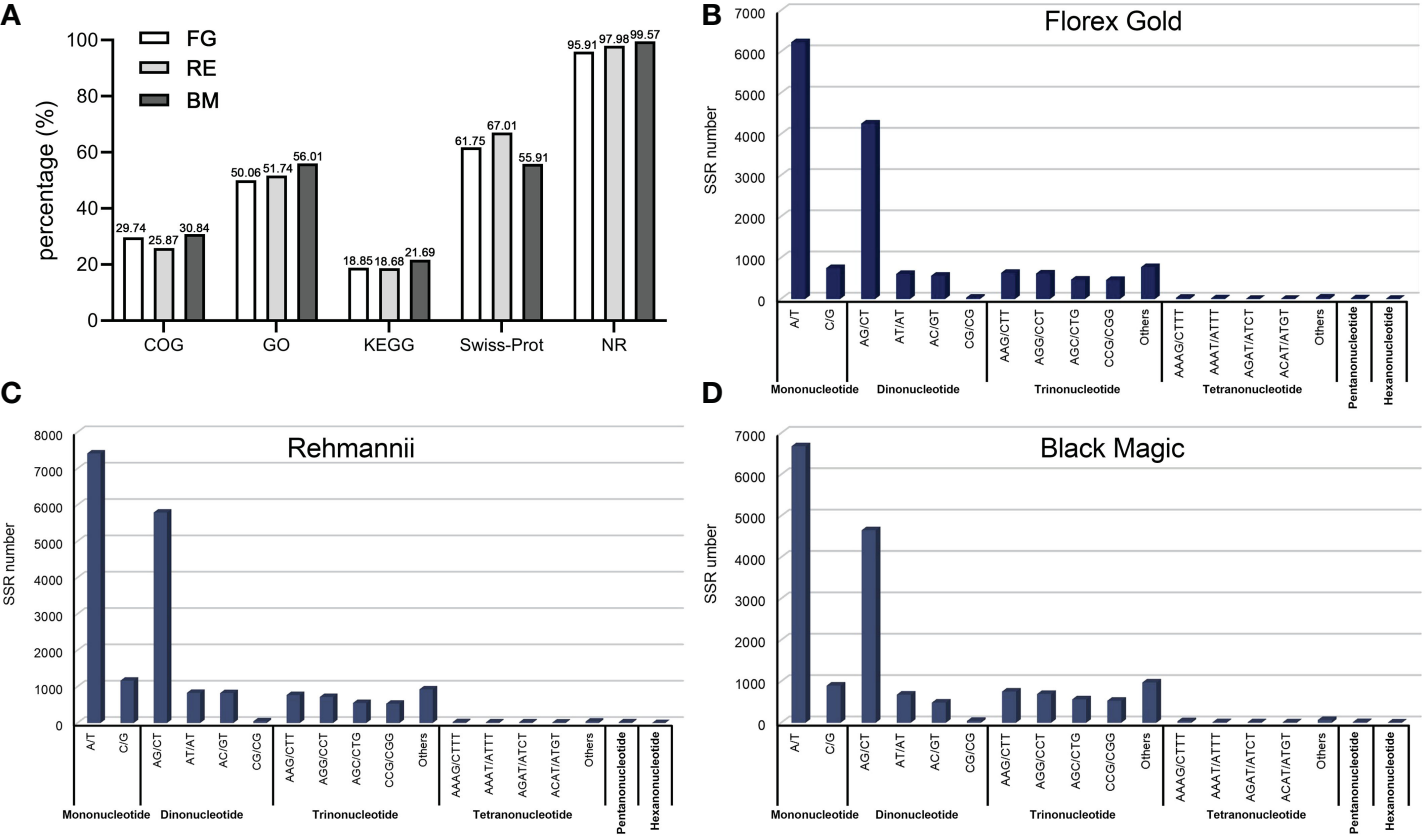
Detailed functional annotations and SSR loci identification in three varieties’ transcriptome assembled sequences. **(A)** Functional annotation of unigenes of Florex gold, Rehmannii and Black Magic; SSR motif types in unigenes of Florex gold **(B)**, Rehmannii **(C)**, and Black Magic **(D)**.

Additionally, the unigenes of each variety were aligned to the COG, GO, KEGG, Swiss-Prot, and NR databases using BLASTX and HEMMER with E-values below 10E-5 and 10E-10, respectively. As shown in **Figure 1A** and **Supplementary Figure S1**, it was found that 33,419, 29,992, and 55,420 unigenes of each variety could be annotated by these five public databases, accounting for 30.58%, 33.39%, and 45.86% of all unigenes, respectively. The NR database for each variety had the most annotated information, with 32,053, 29,386 and 55,183 unigenes, respectively, accounting for 29.33%, 32.71% and 45.67% of all sequences. In contrast, KEGG had the least annotated information, with 6,299, 5,602, and 12,022 unigenes, which respectively represented 5.76%, 6.24%, and 9.95% of all unigenes. It was expected that the number of annotations collected for unigenes with a length greater than or equal to 1000 bp would be the highest in each variety.

### 3.2 Identification of SSR loci in unigenes derived from three-varietal transcriptome

It was determined that among the 15,938, 12,750, and 14,151 unigenes in Florex Gold, Rehmannii, and Black Magic, a total of 19,753, 15,526, and 17,160 SSR loci were identified, respectively. Of these, 3000, 2227, and 2428 unigenes contained at least one candidate SSR locus. In addition to that, 1335, 992, and 1092 SSR loci were found to have varied motif variation patterns with each variety. In terms of SSR frequency, with each kind of variety, one SSR locus was predicted every 3.4 kb, 3.5 kb, and 4.1 kb, respectively (**Supplementary Table S3**). Based on the distribution of SSR motifs, it has been determined that, except for mononucleotide SSR loci, dinucleotide motifs were the most common, followed by trinucleotides, which have been identified in Florex Gold, Rehmannii, and Black Magic, respectively, with 7,499, 5,464, and 5,869, as well as 3,504, 2,964, and 3,527. The other motif types were in lesser numbers. As illustrated in **Figure 1A**, AG/CT is the most prevalent dinucleotide motif type (5,794, 4,257, and 4,655), followed by AT/AT and AC/GT (825, 565, 489, and 832, 609, 681 for each variety, respectively), while CG/CG was the least abundant (48, 33, 44). In terms of trinucleotide motif types (**Figures 1B–D**), AAG/CTT and AGG/CCT were the most common (771, 636, 755 and 719, 619, 697, respectively), followed by AGC/CTG and CCG/CGG (553, 472, 567 and 533, 462, 533, respectively).

### 3.3 Identification of polymorphism SSR loci in homologous unigenes using the CandiSSR pipeline

**Figure 2:**
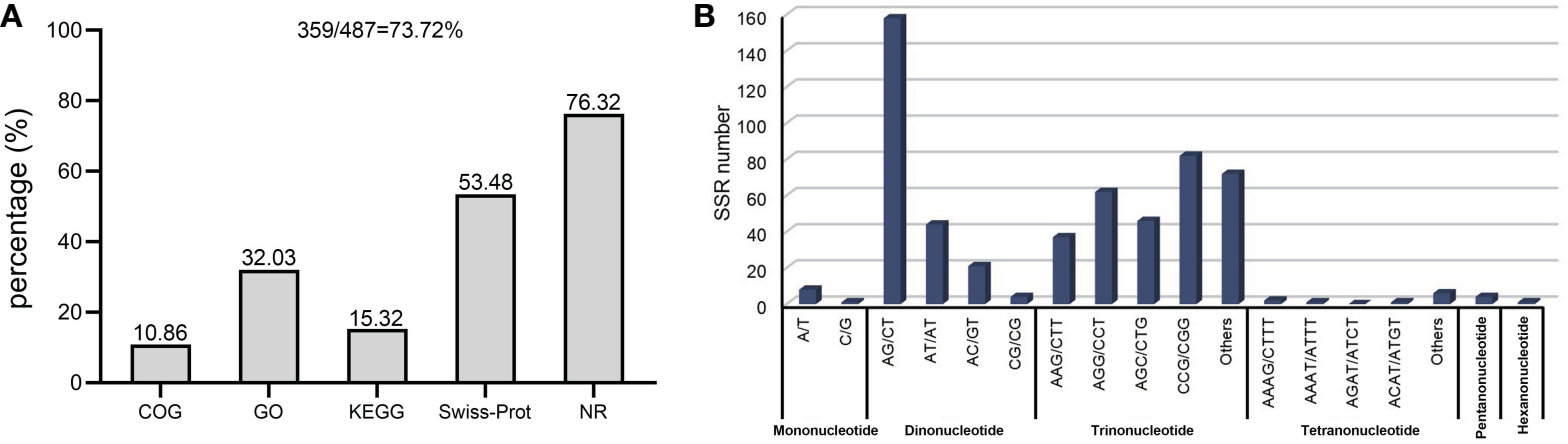
Functional annotation **(A)** and identification of SSR motif **(B)** in transcriptome-derived homology unigenes of three varieties though CandiSSR pipeline.

We defined the unigenes acquired from Florex Gold as the reference sequence and the other two as the query sequence, based on factors such as the amount of transcriptome data and the length of the assembled unigenes, before using the CandiSSR pipeline to identify polymorphic SSR loci. From 487 homologous unigenes, 550 potential SSR loci were monitored (**Supplementary Table S3**). Of these, 57 unigenes displayed at least one SSR locus. There are, however, only two SSRs present in the compound motif pattern. As detected in the above-mentioned SSR motif type in three varieties’ transcriptome unigenes, the polymorphic SSR identified here (**Figure 1A**) also revealed that AG/CT (158) was the most prevalent dinucleotide motif followed by AT/AT (44) and AC/GT (21). The least common di-motif was CG/CG (4). The most frequent trinucleotide motifs in **Figure 2B** are the CCG/CGG (82), the AGG/CCT (62), the AGC/CTG (46), and the AAG/CTG (37).

**Figure 3:**
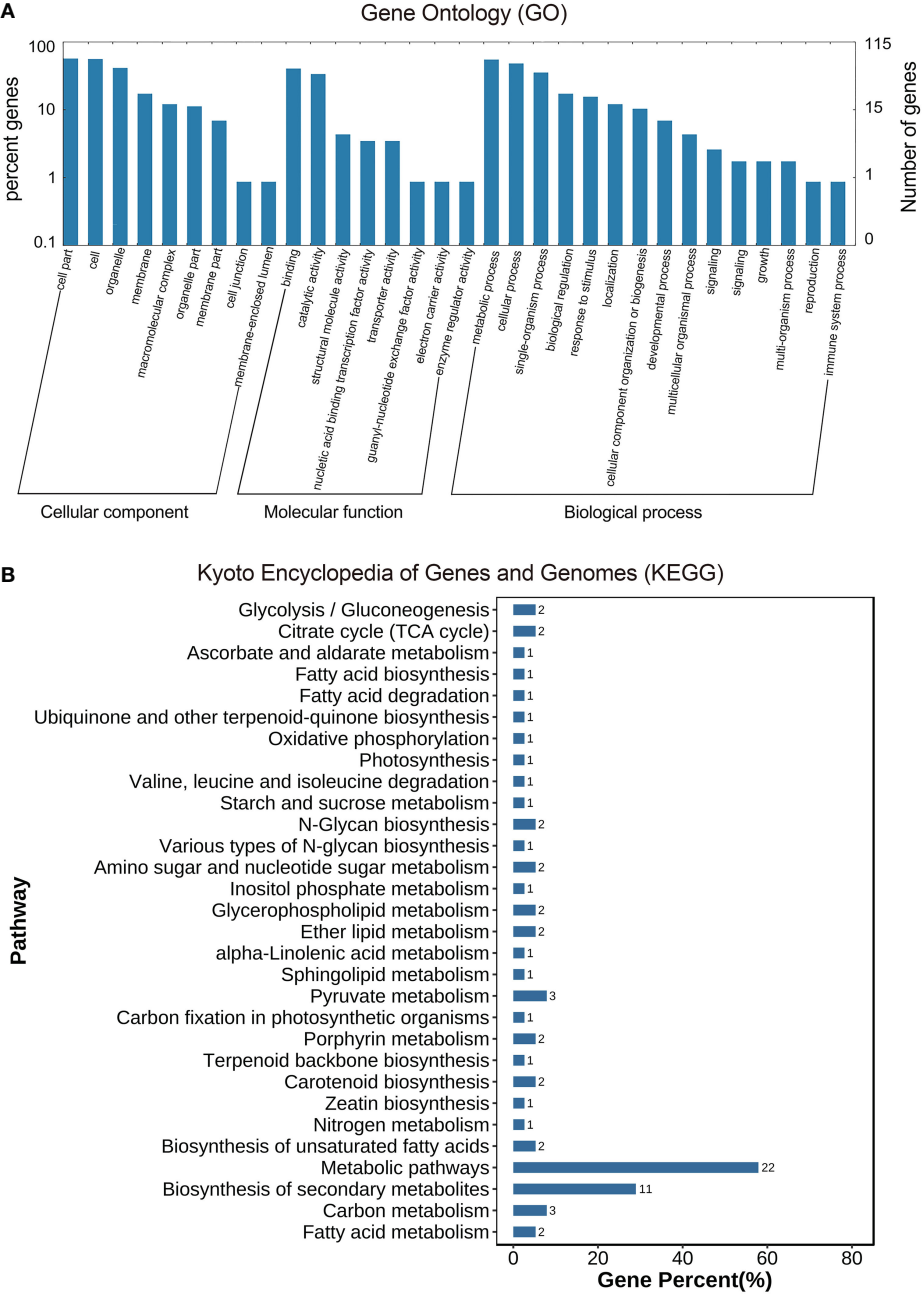
Homology search of the transcriptome-derived 359 unigenes of the three varieties, Florex gold (FG), Rehmannii (RE) and Black Magic (BM) 359 unigenes against the GO database **(A)** assignment into biological process, molecular function and cellular component at the third level, and KEGG database **(B)**.

**Figure 4:**
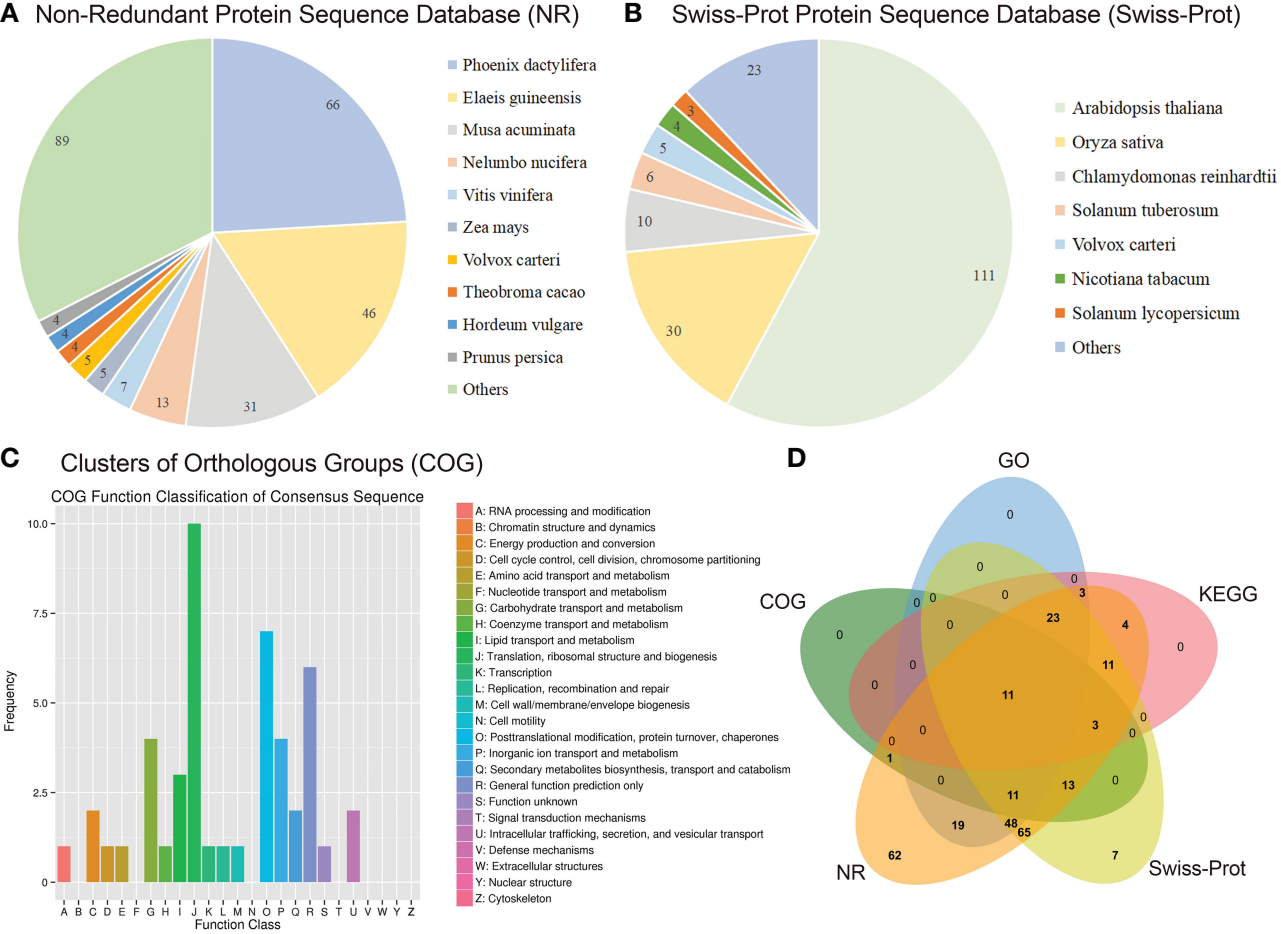
Homology search of the transcriptome-derived 359 unigenes of the three varieties, of Florex gold (FG), Rehmannii (RE) and Black Magic (BM) against NR, Swiss-Prot and COG database. The distribution of homologous species with signicant hit in NR **(A)** and Swiss-Prot **(B)**. **(C)** COG function classification of above consensus unigenes. **(D)** Venn diagram shows the functional annotation of FG-RE-YN consensus unigenes.

As described previously, all 487 homologous unigenes containing EST-SSR loci were searched against multiple databases to extract as much annotated information as possible (**Supplementary Table S3**). Of the 359 unigenes which were annotated, 274 (76.32%) showed a significant similarity to known proteins in the NR database. COG is 39 (10.86%); GO is 115(32.03); KEGG is 55(15.32%), Swiss-Prot is 192 (53.46%) (**Figure 2A**; **Supplementary Table S4**). **Figure 3A** illustrates GO terms for biological processes, cellular components, and molecular functions. In **Figure 3B**, metabolic pathways are most enriched in KEGG pathways (22) followed by biosynthesis of secondary metabolites (11). A number of homologous species hit for those annotated unigenes in the NR database (**Figure 4A** and **Supplementary Table S4**) were found for *Phoenix dactylifera* (66) and *Elaeis guineensis* (46), followed by *Musa acuminata* (31), *Nelumbo nucifera* (13), *Vitis vinifera* (7), *Zea mays* (5), *Volvox carter* (5), *Theobroma cacao* (4), *Hordeum vulgare* (4), and *Prunus persica* (4). The homologous hits were also represented in the Swiss-Prot database, but with different species. Annotated unigenes with the highest frequency in COG categorization included translation, ribosome structure, and biogenesis, as shown in **Figure 4C**. **Figure 4D** further illustrates the overlapped annotated unigenes across five databases. In light of these functional annotations, it appears that homologous unigenes containing SSR loci may be superior for use in future functional molecular marker-assisted breeding of calla lily.

### 3.4 Genetic diversity of polymorphism EST-SSR markers in 160 accessions of colored calla lily

Among the 550 consensus SSR loci in three varieties, 460 loci with suitable flanking sequences were selected for primer pair design. A total of 200 primer pairs were then selected at random for preliminary verification across the 21 accessions previously used in our study (Wei et al., 2016). According to our previous research based on the same accessions, out of the 200 primer pairs developed from a single transcriptome assembled unigene of Rehmannii, 139 primer pairs could amplify bands, whereas only 58 primer pairs were polymorphic. The present study found that 181 primer pairs could amplify bands, while the number of polymorphic primers nearly doubled to 115 as compared to the previous study (**Supplementary Table S6**). It appears that the EST-SSR markers obtained by using the CandiSSR algorithm based on multiple transcriptomes are more efficient and polymorphic in comparison with our previous study.

To determine the diversity of 160 colored calla lilies (**Supplementary Tables S1**, **S7**, **S8** and **Supplementary Figure S2**), 40 pairs of EST-SSR markers were randomly screened, and 109 alleles were detected (**Table 1**). The number of loci for each allele locus varied from 2 to 5. It is estimated that the average number of *Na* is 2.73. The average number of *Ne* is 1.79, with the highest number occurring at locus Zacp158 (2.88) and the lowest at locus Zacp134 (1.07). With an average of 0.63, the *I* was highest at Zacp169 (1.09), and lowest at Zacp134 (0.15). It has also been found that the genetic diversity level is correlated with *Ho* as well. The locus with the highest *Ho* was Zacp110 (0.94), and the lowest was Zacp14 (0.06), with an average of 0.40. As far as the *He* is concerned, the highest locus was Zacp158 (0.66), while the lowest loci were Zacp14 (0.07) and Zacp134 (0.07). The mean *Nei* for the entire collection was 0.39. Of the loci in the *PIC*, Zacp158 had the highest value (0.67), while Zacp134 had the lowest value (0.06).

**Table 1:** Descriptive statistics of the 40 ESR-SSRs markers scored on 160 colored calla lily accessions.

Locus	*Na*	*Ne*	*I*	*Ho*	*He*	*Nei*	*PIC*
Zacp3	3	1.89	0.79	0.57	0.47	0.47	0.42
Zacp9	2	1.95	0.68	0.43	0.49	0.49	0.56
Zacp14	2	1.08	0.16	0.06	0.07	0.07	0.21
Zacp15	2	1.12	0.22	0.10	0.11	0.11	0.17
Zacp17	2	1.19	0.29	0.17	0.16	0.16	0.31
Zacp18	2	1.15	0.25	0.14	0.13	0.13	0.34
Zacp25	2	1.96	0.68	0.38	0.49	0.49	0.57
Zacp26	3	1.13	0.25	0.13	0.12	0.12	0.11
Zacp30	2	2.00	0.69	0.19	0.50	0.50	0.42
Zacp32	3	1.81	0.69	0.54	0.45	0.45	0.36
Zacp40	2	1.20	0.31	0.18	0.17	0.17	0.22
Zacp49	2	1.10	0.19	0.09	0.09	0.09	0.41
Zacp59	3	2.12	0.83	0.47	0.53	0.53	0.44
Zacp69	4	2.34	0.97	0.54	0.57	0.57	0.60
Zacp74	3	2.25	0.89	0.83	0.56	0.55	0.49
Zacp79	2	1.21	0.32	0.20	0.18	0.18	0.17
Zacp80	3	1.87	0.79	0.63	0.47	0.47	0.41
Zacp82	4	1.85	0.72	0.67	0.46	0.46	0.38
Zacp88	3	2.05	0.75	0.71	0.51	0.51	0.41
Zacp93	2	1.81	0.64	0.68	0.45	0.45	0.35
Zacp95	3	2.16	0.84	0.38	0.54	0.54	0.59
Zacp97	2	1.11	0.20	0.10	0.10	0.10	0.10
Zacp103	2	1.46	0.49	0.39	0.32	0.31	0.43
Zacp104	2	1.96	0.68	0.81	0.49	0.49	0.38
Zacp110	4	2.59	1.05	0.94	0.62	0.61	0.53
Zacp112	2	1.98	0.69	0.14	0.50	0.50	0.38
Zacp115	5	2.13	0.95	0.39	0.53	0.53	0.62
Zacp118	2	1.46	0.49	0.22	0.32	0.31	0.34
Zacp125	3	2.01	0.71	0.63	0.50	0.50	0.55
Zacp126	2	1.18	0.28	0.16	0.15	0.15	0.34
Zacp132	4	2.49	1.05	0.20	0.60	0.60	0.63
Zacp133	3	2.46	0.97	0.82	0.60	0.59	0.59
Zacp134	2	1.07	0.15	0.07	0.07	0.07	0.06
Zacp135	3	1.92	0.76	0.41	0.48	0.48	0.42
Zacp158	3	2.88	1.08	0.79	0.66	0.65	0.67
Zacp162	2	1.95	0.68	0.36	0.49	0.49	0.46
Zacp164	2	2.00	0.69	0.32	0.50	0.50	0.52
Zacp165	3	1.56	0.56	0.32	0.36	0.36	0.39
zacp169	5	2.36	1.09	0.32	0.58	0.58	0.61
zacp171	4	1.81	0.76	0.52	0.45	0.45	0.41
Mean	2.73	1.79	0.63	0.40	0.40	0.39	0.41

Na, number of alleles per locus; Ne, the effective number of alleles; I, Shannon’s Information index; Ho, observed heterozygosity; He, expected heterozygosity; Nei, Nei’s expected heterozygosity; PIC, polymorphism information content.

### 3.5 Construction of core germplasm using the polymorphism EST-SSR markers

Core Hunter software was utilized by setting the weights of MR and SH to 0.5 each and sampling proportions of 10%, 15%, 20%, 30%, 40%, and 50%, which resulted in core germplasm samples of 16, 24, 32, 40, 48, 64, and 80, respectively. The sampling proportion in SA, which consists of SANA method-PM (SA) and SAGD method-PM (SD), was the same as in Core Hunter software. In **Table 2**, diversity parameters derived from 40 EST-SSR markers are summarized for each sampled core collection. Those parameters of both SA and SD reached their peak when the number of core germplasm reached 48 (30%), but with a difference that declined in the former at 64 (40%) while in the latter at 48 (30%). Similarly, the highest genetic parameters were found in the Core Hunter when the sampled number reached 40 (25%), but began to decline at 48 (30%).

**Table 2:** Variability parameters for colored calla lily with the different core subsets.

Construction method	Sampling ratio(%)	Number of germplasms	*Na*	*Ne*	*I*	*Ho*	*He*	*Nei*	*PIC*
Initial germplasm		160	109	1.79	0.63	0.40	0.40	0.39	0.41
	50%	80	107	1.78	0.62	0.39	0.39	0.39	0.40
	40%	64	106	1.76	0.61	0.38	0.39	0.39	0.40
	30%	48	106	1.78	0.62	0.38	0.40	0.39	0.41
SANA method-PM(SA)	25%	40	102	1.77	0.62	0.38	0.39	0.39	0.40
	20%	32	101	1.77	0.62	0.37	0.40	0.39	0.40
	15%	24	94	1.78	0.60	0.37	0.39	0.39	0.38
	10%	16	92	1.78	0.60	0.36	0.40	0.39	0.38
SAGD method PM(SD)	50%	80	107	1.79	0.63	0.41	0.40	0.40	0.41
	40%	64	105	1.79	0.63	0.42	0.40	0.40	0.40
	30%	48	105	1.79	0.64	0.42	0.40	0.40	0.41
	25%	40	104	1.80	0.63	0.38	0.40	0.40	0.41
	20%	32	101	1.79	0.62	0.39	0.40	0.39	0.40
	15%	24	100	1.76	0.61	0.37	0.40	0.38	0.40
	10%	16	88	1.75	0.57	0.37	0.39	0.37	0.36
Core Hunter	50%	80	108	1.82	0.65	0.42	0.40	0.40	0.40
	40%	64	107	1.81	0.64	0.42	0.40	0.40	0.40
	30%	48	107	1.81	0.64	0.42	0.40	0.40	0.40
	25%	40	107	1.82	0.65	0.41	0.41	0.40	0.40
	20%	32	105	1.82	0.64	0.43	0.41	0.40	0.40
	15%	24	99	1.80	0.62	0.39	0.40	0.39	0.39
	10%	16	92	1.79	0.60	0.39	0.40	0.38	0.39
Mp method (Power Core)	26.25%	42	109	1.83	0.66	0.37	0.40	0.40	0.43

Na, observed number of alleles; Ne, the effective number of alleles; I, Shannon’s Information index; Ho, observed heterozygosity; He, expected heterozygosity; Nei, Nei’s expected heterozygosity; PIC, polymorphism information content.

**Figure 5:**
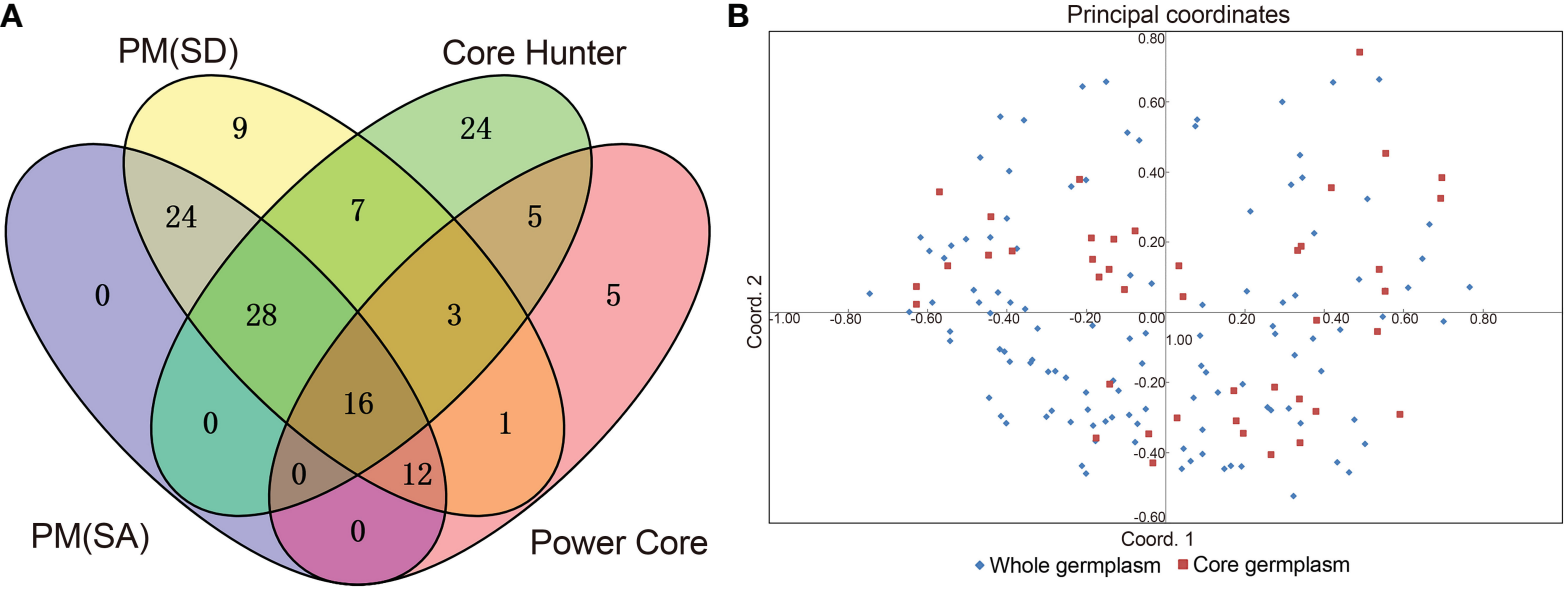
Venn diagram and Principal component plot. **(A)** Venn diagram of core collections defined by four methods, including SANA method-PM (SA) and SAGD method-PM (SD), Core Hunter, and Power Core (Mp method), and **(B)** Principal component plot between the core and entire collections based on 40 polymorphism EST-SSR molecular marker.

However, it is not necessary to pre-set the Power Core software in this manner. Using the Mp (maximization Power Core) method, all genetic parameters were estimated better than described above when the sample size was 42 (26.25%), which captures all the alleles identified in 160 germplasms, thus allowing us to select those 42 accessions for further analysis. **Table 3** illustrates that there were no significant differences (P > 0.05) in genetic diversity parameters between the 42 core accessions and 160 original germplasms. **Figure 5A** also provides insight into the distance between individuals as determined by principal coordinates analysis. There were 42 core germplasms distributed evenly across the whole 160 samples, which showed that they had been well preserved. Therefore, it can be stated that the M strategy is an efficient way to restore all alleles of the entire collection in order to reduce redundancy while capturing most of the genetic diversity (Marita et al., 2000; Kim et al., 2007). In addition to that, **Figure 5B** shows that a total of sixteen accessions are shared by above four methods, indicating that these accessions are all considered to be important to the study. 16 accessions maybe super representative of the core accession (**Supplementary Table S9**).

**Table 3:** Comparison of genetic parameters between core collection and original germplasm GraphPad Prism 8.4.3 (GraphPad Software Inc., USA: http://www.graphpad.com/) was used to statistical analysis of P value.

Parameters	Original germplasm	Core germplasm	T-test
*Na*	2.73	2.73	>0.9999
*Ne*	1.79	1.83	0.71
*I*	0.63	0.66	0.73
*Ho*	0.40	0.63	0.53
*He*	0.40	0.40	0.83
*Nei*	0.39	0.40	0.93
*PIC*	0.41	0.43	0.50

*P < 0.05; **P < 0.01; ***P < 0.001; ****P < 0.0001. T-test T in table does not reach a significant level.

**Figure 6:**
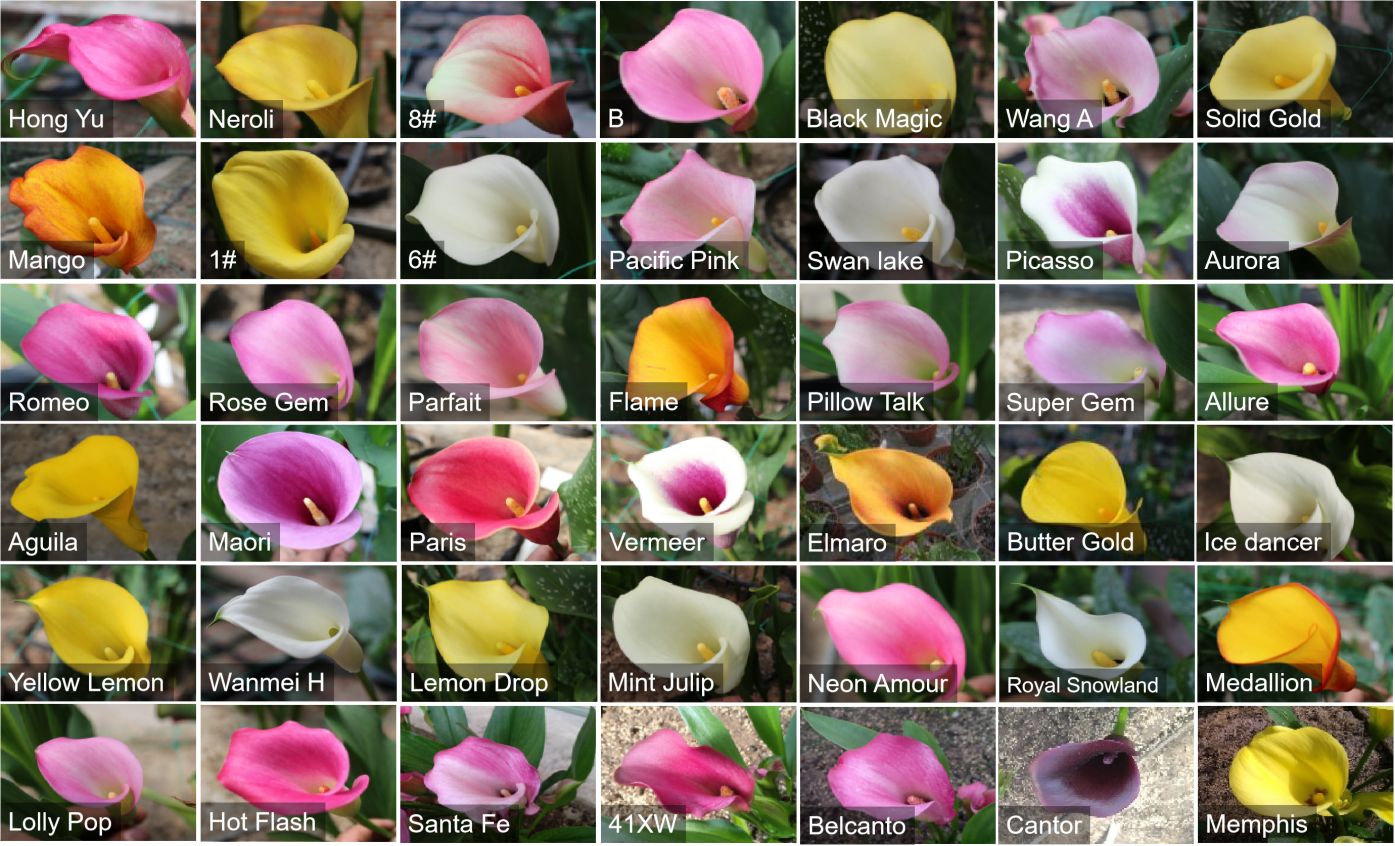
Total 42 core germplasms of colored calla lily defined by the software Power Core.

A summary of the final selection of primary core germplasm for colored calla lily was provided in **Supplementary Table S10** and **Figure 6**. The 42 accessions were grouped into five series based on the color of their spathes. There were 12 germplasm samples in the pink series (B, Wang A, Pacific Pink, Aurora, Rose Gem, Parfait, Pillow Talk, Super Gem, Neon Amour, Lolly Pop, Hot Flash, Santa Fe). The series purple and yellow includes ten accessions (Hong Yu, Picasso, Romeo, Allure, Maori, Paris, Vermeer, 41XW, Belanto, Cantor) and eight accessions (Black Magic, Solid Gold, 1#, Aguila, Butter Gold, Yellow Lemon, Lemon Drop, Memphis), while the white and orange series respectively consist of six accessions (6#, Swan Lake, Ice Dancer, Wanmei H, Mint Julip, Royal Snowland; and Neroli, 8#, Mango, Flame, Elmaro, Medallion). These screened materials will allow us to breed novel colored calla lily more effectively in the future.

## 4 Discussion

DNA markers, particularly SSR, are a reliable and effective technique for identifying germplasm resources in several research (Zhang et al., 2011). As a codominant marker, EST-SSRs are derived from transcribed DNA regions, the majority of which are present in functional genes, indicating that they are associated with important phenotypes. Consequently, they offer irreplaceable potential benefits for interspecies transfer and more generally consistent amplification efficiency (Gupta et al., 2003). There are now some research implementations using EST-SSRs in calla lily (Wei et al., 2016; Wei et al., 2017). EST-SSRs, however, are derived from relatively conserved coding regions of genes, resulting in low polymorphism. Thus, it is challenging to rapidly and efficiently screen thousands of SSR sites for functional and polymorphic candidate markers. To address the above challenges, many researchers have proposed the use of bioinformatics to identify potential candidate polymorphic EST-SSRs and to design primers for their validation. A pipeline for identifying candidate polymorphic SSRs from assembled transcriptome sequences has been developed (Xia et al., 2016). By using CandiSSR pipeline, we obtained optimized EST-SSRs from three assembled unigenes of colored calla lily, which provides an efficient method of developing EST-SSRs for multi-varietal transcriptomes and is an effective tool for developing SSR markers and marker-assisted breeding in plants lacking a reference genome. However, we observed only 109 alleles in 160 accessions of colored calla lily, with a number of alleles per locus (*Na*) ranging from 2 to 5 per locus and an average value of 2.73. In contrast, in our previous study (Wei et al., 2017) of the diversity and population structure of 117 accessions using transferable EST-SSR markers from white calla lily (*Z. aethiopica*), 111 alleles were detected in 34 EST-SSR loci, and their number of alleles per locus ranged from 2 to 10, with an average value of 3.58. Considering the collected materials are almost identical, we speculated that the polymorphic EST-SSR markers obtained using the CandiSSR pipeline may lose some genetic diversity due to homologous sequences being derived from each variety and being more conserved.

It has been proposed to develop core germplasm to improve the use of genetic resources in breeding programs. Core germplasm is a subset of accessions from the entire collection that encompasses the majority of the genetic diversity of the species, which may be examined in depth, and the information derived from the assessment can be used to guide the effective utilization of the entire collection (Brown, 1989). While more and more germplasms are emerging, conservation and management of them become more difficult than ever (Hintum et al., 2000). Calla lilies and other bulbs, especially those grown under difficult conditions, are subject to weakening bulbs and plants, as well as deteriorating quality. In addition, some varieties are severely degraded during cultivation due to improper management, pests, and diseases. A core germplasm bank with fewer costs associated with germplasm manipulation and maintaining maximum genetic diversity (Frankel and Brown, 1984) is therefore advocated as being very essential (Pessoa-Filho et al., 2010).

Core collection are a vital component of plant breeding that can enhance the efficiency with which germplasm resources are utilized for plant genetic improvements and new variety development. As an important determinant of the result of the construction of the core germplasm, the sampling strategy can not only reduce genetic redundancy but also preserve its genetic diversity to the greatest possible extent. Generally, it is divided into two categories, random sampling and stratified sampling, with the latter being regarded as significantly superior to the former in many studies (Brown, 1989; Casler, 1995; Escribano et al., 2008). There are a number of sampling methods that can be used in stratified sampling, including proportional, logarithmic, square root, and genetic diversity, each of which has its own advantages and disadvantages. It is important to consider information such as the quantity of germplasm materials, grouping, and genetic diversity when choosing a sampling strategy. Using 40 transcriptome-derived homologous EST-SSR markers, the strategy M (Power Core), simulated annealing algorithm (SA; SANA), simulated annealing algorithm (SA; SAGD) and Core Hunter were respectively used to define the preliminary core collection of colored calla lily. Utilizing the M strategy in Power Core, the most suitable subset of core germplasms from 160 colored calla lilies was identified as 42 core germplasms. M strategy, incorporating the maximization of alleles (*Na*) and the genetic diversity, automatically generates a reasonable sampling ratio through software such as Power Core, which is scientifically and reliably accurate (Gouesnard et al., 2001; Kim et al., 2007; Escribano et al., 2008). Additionally, it has been recognized as a totally new methodology distinct from other approaches, which simplifies the process of generating a core set while significantly reducing the number of core entries, while retaining 100% of the diversity as categorical variables. Hence, we believe that the core collection defined here represents all genetic diversity in the original germplasm.

Core germplasm aims to represent the genetic diversity of original germplasm to the greatest extent possible using the fewest resources. The most critical aspect of achieving this goal is the selection of an appropriate sampling ratio. To date, different plant germplasms have been constructed at home and abroad to a proportion of 5% to 40% (Wang et al., 2007; Escribano et al., 2008; Liang et al., 2014; Wang et al., 2014). All studies, however, failed to provide a unified sampling rate, which is mainly due to differences in the degree of collection, level of genetic diversity, and the structure of the internal genetics of different plant resources. It is estimated that 26.25% of the germplasm in this study consists of core germplasm, and it has restored all alleles of 160 germplasms. A principal coordinate analysis (**Figure 5A**) revealed a genetic relationship between the core germplasm (42) and the whole germplasm (160), which further confirmed the representativeness of the current core germplasm. Several evaluation parameters, such as *Na*, *I*, and *Nei*, should be taken into consideration when determining whether the core accession is representative. While the *Na* is considered to be the most relevant indicator (Song et al., 2014; Pereira-Lorenzo et al., 2018; Nie et al., 2021), other metrics, including *PIC*, *Ne*, may also be used. Moreover, in an ideal core population, heterozygosity, defined as the probability that two randomly selected samples within a population have different alleles, should be roughly equivalent to that of the original germplasm. As a measure of the representativeness of the core germplasm, multiple parameters, such as *Na*, *Ne*, *I*, *Ho*, *He*, *Nei*, and *PIC*, were therefore considered. We found (**Table 3**) that these genetic parameters in the 42 core germplasms constructed in this study were excellent, which indicated a good effect on the preservation of genetic diversity. It is expected that the initial core germplasm collection, consisting of 42 accession types, will provide a valuable breeding resource for improving breeding efficiency and accelerating the development of new varieties of colored calla lily in the future.

## Data availability statement

The original contributions presented in the study are publicly available. This data can be found here: NCBI, PRJNA883926 and PRJNA316785; NGDC, PRJCA000375.

## Author contributions

ZW and XiW conceived the study. YW, XuW, XS, HL, DW and HW collected materials and performed experiments. YW and TY analyzed data and drafted the manuscript. ZW, TY, XiW, YL and GZ revised the manuscript. All authors contributed to the article and approved the submitted version.

## Supplementary material

Supplementary Figure 1 The annotated proportion of transcriptome-derived unigenes of variety, Florex gold (FG), Rehmannii (RE), and Black Magic (BM).

Supplementary Figure 2 Polymorphic polyacrylamide gel diagram.

Supplementary Table 1 The 160 accessions of colored calla lily.

Supplementary Table 2 The transcriptome-derived unigenes of three colored calla lily variteies.

Supplementary Table 3 SSR frequency and motif type in transcriptome-derived unigenes of three colored calla lilyvarieties.

Supplementary Table 4 Integrated functional annotation and classification for transcriptome unigenes of three colored calla lily varieties.

Supplementary Table 5 Primer sequences of 460 EST SSRs used for marker validation.

Supplementary Table 6 Primer sequences of 200 EST SSRs used for marker validation.

Supplementary Table 7 Primer sequences of 40 EST SSRs used for marker validation.

Supplementary Table 8 The raw data of 40 EST-SSRs tested in 160 colored calla lilies.

Supplementary Table 9 The 16 core accessions of colored calla lily with intersection of the four methods.

Supplementary Table 10 The 42 core accessions of colored calla lily.
