# Supplementary figures and images for: Develop a preliminary core germplasm with the novel polymorphism EST-SSRs derived from three transcriptomes of colored calla lily (Zantedeschia hybrida)
Source: Front Plant Sci. 2023 Feb 2;14:1055881. doi: 10.3389/fpls.2023.1055881 (PMC9933510; doi:10.3389/fpls.2023.1055881)

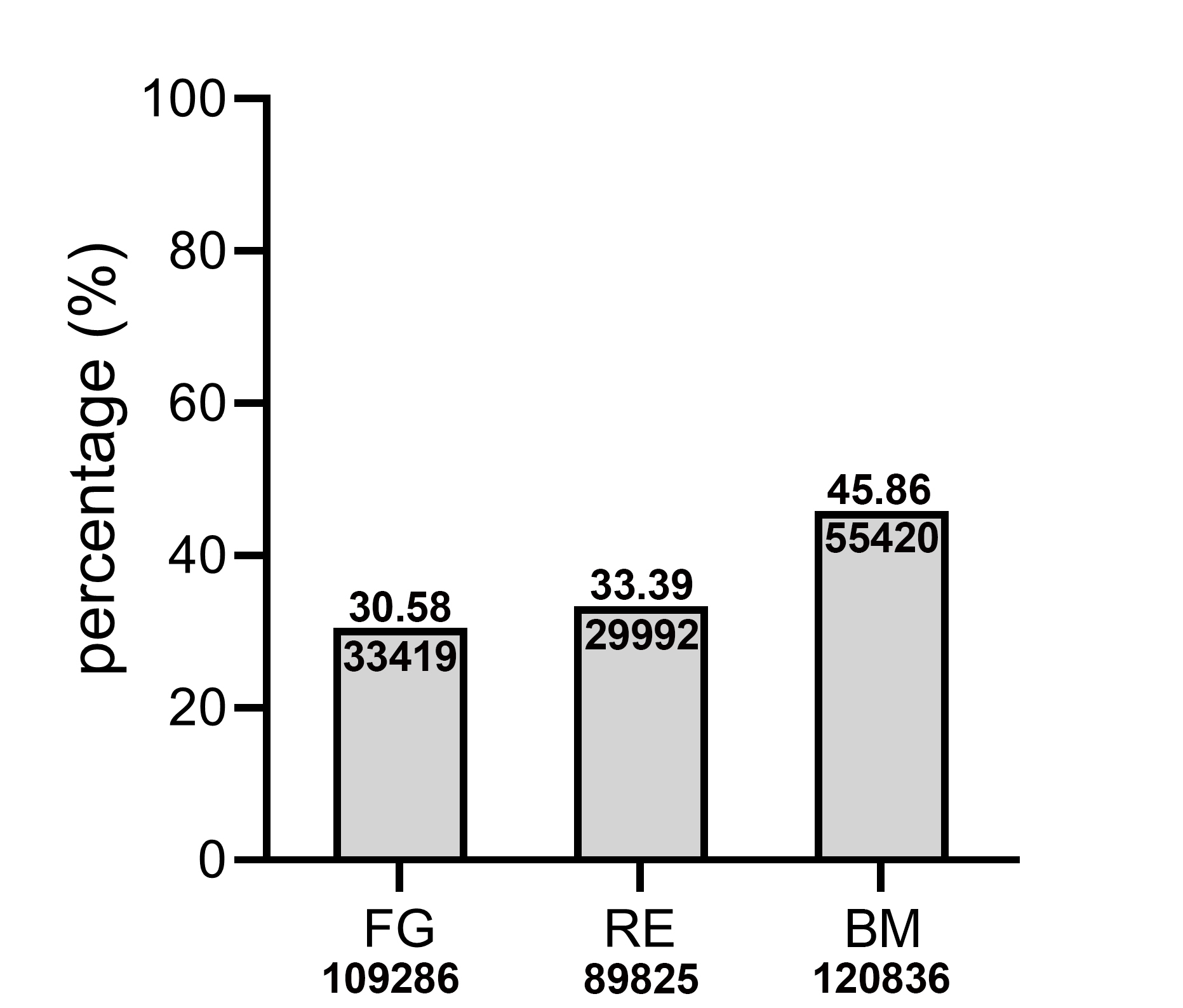

Supplement: Supplementary Figure 1 — The annotated proportion of transcriptome-derived unigenes of variety, Florex gold (FG), Rehmannii (RE), and Black Magic (BM). [file Image_1.jpeg]
