# Supplementary material for: Develop a preliminary core germplasm with the novel polymorphism EST-SSRs derived from three transcriptomes of colored calla lily (Zantedeschia hybrida)
Source: Front Plant Sci. 2023 Feb 2;14:1055881. doi: 10.3389/fpls.2023.1055881 (PMC9933510; doi:10.3389/fpls.2023.1055881)
Supplement: Supplementary Table 1 — The 160 accessions of colored calla lily. [file Table_1.docx]

| **Accessions** | **Country** | **Color** | **Leaves** | **Use Type** |
| --- | --- | --- | --- | --- |
| Hong Baoshi | USA | Pink | Lanceolate, not spotted | Pot-flower |
| Hong Yu | USA | Purple | Ovate, not spotted | Pot-flower |
| Mei Yu | USA | Purple | Lanceolate, not spotted | Pot-flower |
| Xiangfeng Red | Unkown | Red | Hastate, spotted | Pot-flower |
| Majestic Red | New Zealand | Red | Lanceolate, not spotted | Pot-/Cut-flower |
| Neroli | New Zealand | Orange | Hastate, spotted | Pot-/Cut-flower |
| Xiangyuan Red | New Zealand | Red | Ovate, spotted | Pot-/Cut-flower |
| V# | USA | Purple | Hastate, spotted | Pot-flower |
| Gold Affair | New Zealand | Yellow | Hastate, spotted | Pot-/Cut-flower |
| 8# | Unkown | Orange | Saggitate, spotted | Pot-/Cut-flower |
| Hot shot | New Zealand | Orange | Hastate, spotted | Cut-flower |
| Pot of Gold | New Zealand | Yellow | Saggitate, spotted | Pot-/Cut-flower |
| B-Y | USA | Yellow | Saggitate, spotted | Pot flower |
| Florex Gold | New Zealand | Yellow | Hastate, spotted | Cut-flower |
| Da Huang | USA | Yellow | Saggitate, spotted | Pot-/Cut-flower |
| Wan Mei | USA | Pink | Ovate, spotted | Pot-/Cut-flower |
| Yang Guang | USA | Yellow | Saggitate, spotted | Pot-flower |
| Huo Yan | USA | Yellow | Saggitate, spotted | Pot-flower |
| B | Unkown | Pink | Ovate, spotted | Pot-flower |
| 9# | New Zealand | Yellow | Saggitate, spotted | Cut-flower |
| Black Magic | New Zealand | Yellow | Saggitate, spotted | Cut-flower |
| Yellow | USA | Yellow | Saggitate, spotted | Pot-flower |
| Crystal Blush | New Zealand | White | Lanceolate, not spotted | Pot-/Cut-flower |
| Wang A | New Zealand | Pink | Ovate, spotted | Pot-flower |
| Zi Yu | USA | Pink | Ovate, not spotted | Pot-flower |
| Pink Persuasion | New Zealand | Pink | Ovate, spotted | Pot-/Cut-flower |
| Solid Gold | New Zealand | Yellow | Saggitate, spotted | Cut-flower |
| YN | New Zealand | Yellow | Saggitate, spotted | Cut-flower |
| Harmany | New Zealand | White | Saggitate, spotted | Cut-flower |
| Mango | New Zealand | Orange | Hastate, spotted | Pot-/Cut-flower |
| Ochre | New Zealand | Yellow | Saggitate, spotted | Pot-/Cut-flower |
| 1010# | Unkown | Yellow | Saggitate, spotted | Pot-/Cut-flower |
| Aries | New Zealand | Pink | Hastate, spotted | Pot-/Cut-flower |
| Fen A | New Zealand | Pink | Ovate, spotted | Pot-/Cut-flower |
| 7# | Unkown | Pink | Lanceolate, spotted | Pot-/Cut-flower |
| 4# | Unkown | Yellow | Saggitate, spotted | Cut-flower |
| 1# | Unkown | Yellow | Saggitate, spotted | Cut-flower |
| Z15 | Unkown | Yellow | Saggitate, spotted | Cut-flower |
| Chianti | New Zealand | Pink | Ovate, spotted | Pot-/Cut-flower |
| 6# | Unkown | White | Hastate, spotted | Cut-flower |
| Red Sox | New Zealand | Orange | Hastate, spotted | Pot-/Cut-flower |
| Inca Gold | New Zealand | Yellow | Saggitate, spotted | Pot-/Cut-flower |
| Tasman Gold | New Zealand | Yellow | Saggitate, spotted | Pot-/Cut-flower |
| Scarlet Pimpernel | New Zealand | Red | Ovate, spotted | Pot-flower |
| Pacific Pink | New Zealand | Pink | Hastate, spotted | Cut-flower |
| Passion Fruit | New Zealand | Orange | Lanceolate, spotted | Pot-/Cut-flower |
| Swan lake | New Zealand | White | Saggitate, spotted | Pot-/Cut-flower |
| Picasso | New Zealand | Purple | Saggitate, spotted | Pot-/Cut-flower |
| Aurora | New Zealand | Pink | Lanceolate, spotted | Pot-/Cut-flower |
| Schwarzwalder | New Zealand | Purple | Ovate, spotted | Pot-/Cut-flower |
| Romeo | New Zealand | Purple | Lanceolate, spotted | Pot-/Cut-flower |
| Sunshine | USA | Yellow | Saggitate, spotted | Pot-/Cut-flower |
| Rose Gem | USA | Pink | Lanceolate, not spotted | Pot-flower |
| Rubylite Rose | USA | Pink | Lanceolate, not spotted | Pot-/Cut-flower |
| Reno | Netherlands | Purple | Hastate, spotted | Pot-/Cut-flower |
| Odessa | Netherlands | Purple | Ovate, spotted | Pot-flower |
| Gold Finger | New Zealand | Yellow | Saggitate, spotted | Pot-/Cut-flower |
| Parfait | USA | Pink | Ovate, spotted | Pot-/Cut-flower |
| Rubylite Pink Ice | USA | Pink | Ovate, not spotted | Pot-flower |
| Flame | USA | Orange | Saggitate, spotted | Pot-/Cut-flower |
| Pillow Talk | USA | Pink | Lanceolate, not spotted | Pot-/Cut-flower |
| Apricot Glow | Netherlands | Orange | Hastate, not spotted | Pot-/Cut-flower |
| Cupido | Netherlands | Yellow | Hastate, not spotted | Pot-flower |
| Super Gem | USA | Pink | Lanceolate, not spotted | Pot-flower |
| Lipstick | USA | Pink | Lanceolate, not spotted | Pot-flower |
| Tahiti | New Zealand | Yellow | Hastate, spotted | Pot-/Cut-flower |
| Millennium Gold | USA | Yellow | Saggitate, spotted | Pot-/Cut-flower |
| Allure | Netherlands | Purple | Ovate, spotted | Pot-flower |
| Pink Diamond | USA | Pink | Lanceolate, not spotted | Pot-/Cut-flower |
| Amethyst | USA | Purple | Ovate, spotted | Pot-flower |
| Garnet Glow | Netherlands | Pink | Lanceolate, not spotted | Pot-flower |
| Wanmei-E | USA | Pink | Ovate , spotted | Pot-flower |
| Aguila | Netherlands | Yellow | Saggitate, spotted | Pot-/Cut-flower |
| Branco | Netherlands | Yellow | Hastate, spotted | Pot-flower |
| Camaro | Netherlands | Purple | Hastate, spotted | Pot-flower |
| Chicago | Netherlands | Pink | Hastate, spotted | Pot-/Cut-flower |
| Florida | Netherlands | Red | Hastate, spotted | Pot-/Cut-flower |
| Fuego | Netherlands | Orange | Hastate, spotted | Pot-/Cut-flower |
| Galaxy | Netherlands | Red | Hastate, spotted | Pot-/Cut-flower |
| Kloon | Netherlands | Yellow | Hastate, spotted | Pot-/Cut-flower |
| Maori | Netherlands | Purple | Saggitate, spotted | Pot-/Cut-flower |
| Margarita | Netherlands | White | Ovate, spotted | Pot-/Cut-flower |
| Mercedes | Netherlands | Orange | Ovate, not spotted | Pot-flower |
| Murano | Netherlands | Red | Ovate, spotted | Pot-/Cut-flower |
| Paris | Netherlands | Purple | Hastate, spotted | Pot-/Cut-flower |
| Promise | Netherlands | Purple | Saggitate, spotted | Pot-/Cut-flower |
| Red Alert | Netherlands | Orange | Saggitate, spotted | Pot-flower |
| Rehmannii | Netherlands | Pink | Lanceolate, not spotted | Pot-flower |
| Romance | Netherlands | Pink | Hastate, spotted | Pot-/Cut-flower |
| Rose Queen | Netherlands | Pink | Hastate , not spotted | Pot-flower |
| Serrada | Netherlands | Yellow | Saggitate, spotted | Pot-flower |
| Siberia | Netherlands | White | Ovate, spotted | Pot-/Cut-flower |
| Sirio | Netherlands | Orange | Hastate, spotted | Pot-/Cut-flower |
| Sonora | Netherlands | Yellow | Saggitate, spotted | Pot-/Cut-flower |
| Summer Sun | Netherlands | Yellow | Hastate, spotted | Pot-/Cut-flower |
| Vermeer | Netherlands | Purple | Saggitate, spotted | Pot-/Cut-flower |
| Goldilocks | New Zealand | Yellow | Ovate, spotted | Pot-flower |
| Greta | New Zealand | Purple | Ovate, spotted | Pot-flower |
| 134PP | New Zealand | Pink | Ovate, spotted | Pot-/Cut-flower |
| 134IG | New Zealand | Yellow | Saggitate, spotted | Pot-/Cut-flower |
| Sunbird | New Zealand | Yellow | Hastate, spotted | Cut-flower |
| Treasure | New Zealand | Orange | Hastate, spotted | Cut-flower |
| Sunglow | New Zealand | Red | Ovate, spotted | Pot-flower |
| Fen | New Zealand | Pink | Ovate, spotted | Pot-/Cut-flower |
| Z0 | Netherlands | Yellow | Saggitate, spotted | Cut-flower |
| Elmaro | New Zealand | Orange | Saggitate, spotted | Pot-/Cut-flower |
| Butter Gold | New Zealand | Yellow | Saggitate, spotted | Pot-/Cut-flower |
| Coral passion | USA | Orange | Ovate, not spotted | Pot-/Cut-flower |
| Grape velvet | USA | Purple | Ovate, not spotted | Pot-/Cut-flower |
| Ice dancer | USA | White | Hastate, spotted | Pot-/Cut-flower |
| Nightlife | USA | Purple | Ovate, not spotted | Pot-/Cut-flower |
| Z3 | Unkown | Yellow | Saggitate, spotted | Cut-flower |
| Sunrise | New Zealand | Orange | Saggitate, spotted | Pot-/Cut-flower |
| 27 | Unkown | Yellow | Saggitate, spotted | Pot-/Cut-flower |
| Z1 | Netherlands | Yellow | Saggitate, spotted | Cut-flower |
| Yellow Lemon | USA | Yellow | Saggitate, spotted | Pot-/Cut-flower |
| Wanmei H | USA | White | Ovate, spotted | Pot-flower |
| Lemon Drop | New Zealand | Yellow | Saggitate, spotted | Pot-/Cut-flower |
| Dark Eyes | New Zealand | Pink | Lanceolate, not spotted | Pot-/Cut-flower |
| Intimate Ivory | New Zealand | White | Lanceolate, not spotted | Pot-/Cut-flower |
| Mint Julip | USA | White | Lanceolate, not spotted | Pot-/Cut-flower |
| Sweet Talk | New Zealand | Pink | Lanceolate, not spotted | Pot-/Cut-flower |
| Neon Amour | USA | Pink | Lanceolate, not spotted | Pot-/Cut-flower |
| Super Ivory | New Zealand | White | Lanceolate, not spotted | Pot-/Cut-flower |
| Gold Crown | New Zealand | Yellow | Saggitate, spotted | Pot-/Cut-flower |
| Ruby Sensation | New Zealand | Red | Hastate, spotted | Pot-/Cut-flower |
| Merlot | Netherlands | Purple | Hastate, spotted | Pot-/Cut-flower |
| Hot Cherry | New Zealand | Pink | Hastate, spotted | Pot-/Cut-flower |
| Rosa | Netherlands | Red | Ovate, spotted | Pot-/Cut-flower |
| Royal Sun | Netherlands | Yellow | Hastate, spotted | Pot-flower |
| Royal Princess | Netherlands | Red | Lanceolate, not spotted | Pot-flower |
| Royal Snowland | Netherlands | White | Lanceolate, spotted | Pot-flower |
| Orange Red Star | Netherlands | Orange | Ovate, spotted | Pot-/Cut-flower |
| Amarillo | New Zealand | Yellow | Saggitate, spotted | Pot-/Cut-flower |
| Alpine | USA | White | Hastate, spotted | Pot-/Cut-flower |
| Pink Melody | USA | Pink | Ovate, spotted | Pot-/Cut-flower |
| Strawberry Parfait | USA | Red | Ovate, spotted | Pot-/Cut-flower |
| Golden Chalice | USA | Yellow | Saggitate, spotted | Pot-/Cut-flower |
| Festival | USA | Yellow | Saggitate, spotted | Cut-flower |
| Fire Dancer | USA | Orange | Saggitate, spotted | Pot-/Cut-flower |
| Inferno | USA | Yellow | Saggitate, spotted | Pot-/Cut-flower |
| Medallion | USA | Orange | Saggitate, spotted | Pot-/Cut-flower |
| Fire Glow | USA | Pink | Hastate, spotted | Pot-/Cut-flower |
| Lolly Pop | USA | Pink | Ovate, spotted | Pot-flower |
| Hot Flash | USA | Pink | Lanceolate, not spotted | Pot-flower |
| Callafornia Red | USA | Red | Ovate, not spotted | Pot-/Cut-flower |
| Gold Rush | USA | Yellow | Saggitate, spotted | Pot-/Cut-flower |
| Pinkheart | New Zealand | Pink | Hastate, spotted | Pot-/Cut-flower |
| Ruby Tuesday | New Zealand | Red | Ovate, spotted | Pot-flower |
| Premio | Netherlands | Purple | Hastate, spotted | Pot-/Cut-flower |
| Rialto | Netherlands | White | Ovate, spotted | Pot-/Cut-flower |
| 40SD | Netherlands | Yellow | Hastate, spotted | Pot-/Cut-flower |
| Santa Fe | Netherlands | Pink | Ovate, not spotted | Pot-flower |
| 41XW | Netherlands | Purple | Ovate, not spotted | Pot-flower |
| Belcanto | Netherlands | Purple | Ovate, not spotted | Pot-flower |
| 29ML | Netherlands | Red | Ovate, spotted | Pot-/Cut-flower |
| Paco | Netherlands | Purple | Ovate, spotted | Pot-/Cut-flower |
| Cantor | Netherlands | Purple | Ovate, spotted | Pot-/Cut-flower |
| Trinity | Netherlands | Orange | Hastate, spotted | Pot-/Cut-flower |
| Memphis | Netherlands | Yellow | Ovate, spotted | Pot-/Cut-flower |
