# Supplementary material for: Develop a preliminary core germplasm with the novel polymorphism EST-SSRs derived from three transcriptomes of colored calla lily (Zantedeschia hybrida)
Source: Front Plant Sci. 2023 Feb 2;14:1055881. doi: 10.3389/fpls.2023.1055881 (PMC9933510; doi:10.3389/fpls.2023.1055881)
Supplement: Supplementary Table 2 — The transcriptome-derived unigenes of three colored calla lily variteies. [file Table_2.docx]

| **Unigenes information** | **Florex Gold** | **Rehmannii** | **Black Magic** |
| --- | --- | --- | --- |
| Total number | 109,286 | 89,825 | 120,836 |
| Total length (bp) | 66,853,662 | 54,288,470 | 70,770,260 |
| Length Range (bp) | 200-15,860 | 200-19,422 | 200-16,194 |
| 200-300 (bp) | 51,121(46.78%) | 39,715(44.21%) | 50,586(41.86%) |
| 300-500 (bp) | 25,404(23.25%) | 23,662(26.34%) | 34,994(28.96%) |
| 500-1000 (bp) | 16,301(14.92%) | 13,340(14.85%) | 19,550(16.18%) |
| 1000-2000(bp) | 9,994(9.14%) | 8,150(9.07%) | 9,762(8.08%) |
| >2000 (bp) | 6,466(5.92%) | 4,958(5.52%) | 5,944(4.92%) |
| N50 length (bp) | 1030 | 960 | 825 |
| Mean length (bp) | 611.7 | 604.4 | 585.7 |
| GC content | 45.14% | 45.08% | 46.72% |
