# Supplementary material for: Develop a preliminary core germplasm with the novel polymorphism EST-SSRs derived from three transcriptomes of colored calla lily (Zantedeschia hybrida)
Source: Front Plant Sci. 2023 Feb 2;14:1055881. doi: 10.3389/fpls.2023.1055881 (PMC9933510; doi:10.3389/fpls.2023.1055881)
Supplement: Supplementary Table 3 — SSR frequency and motif type in transcriptome-derived unigenes of three colored calla lilyvarieties. [file Table_3.doc]

| **SSR Feature** | **Florex Gold** | **Rehmannii** | **Black Magic** | **CandiSSR** |
| --- | --- | --- | --- | --- |
| Total number of sequences examined | 109,286 | 89,825 | 120,836 | 487 |
| Total size of examined sequences (bp) | 66,853,662 | 54,286,580 | 70,770,260 | 192,872 |
| Total number of identified SSRs | 19,753 | 15,526 | 17,160 | 550 |
| Number of SSR containing sequences | 15,938 | 12,750 | 14,151 | 487 |
| Number of sequences containing more than 1 SSR | 3,000 | 2,227 | 2,428 | 57 |
| Number of SSRs present in compound formation | 1,335 | 992 | 1,092 | 2 |
