# Supplementary material for: Develop a preliminary core germplasm with the novel polymorphism EST-SSRs derived from three transcriptomes of colored calla lily (Zantedeschia hybrida)
Source: Front Plant Sci. 2023 Feb 2;14:1055881. doi: 10.3389/fpls.2023.1055881 (PMC9933510; doi:10.3389/fpls.2023.1055881)
Supplement: Supplementary Table 9 — The 16 core accessions of colored calla lily with intersection of the four methods. [file Table_9.docx]

| **Accessions** | **Country** | **Color** | **Leaves** | **Use Type** |
| --- | --- | --- | --- | --- |
| Hong Yu | USA | Purple | Ovate, not spotted | Pot-flower |
| Solid Gold | New Zealand | Yellow | Saggitate, spotted | Cut-flower |
| Swan lake | New Zealand | White | Saggitate, spotted | Pot-/Cut-flower |
| Romeo | New Zealand | Purple | Lanceolate, spotted | Pot-/Cut-flower |
| Parfait | USA | Pink | Ovate, spotted | Pot-/Cut-flower |
| Flame | USA | Orange | Saggitate, spotted | Pot-/Cut-flower |
| Pillow Talk | USA | Pink | Lanceolate, not spotted | Pot-/Cut-flower |
| Allure | Netherlands | Purple | Ovate, spotted | Pot-flower |
| Paris | Netherlands | Purple | Hastate, spotted | Pot-/Cut-flower |
| Vermeer | Netherlands | Purple | Saggitate, spotted | Pot-/Cut-flower |
| Butter Gold | New Zealand | Yellow | Saggitate, spotted | Pot-/Cut-flower |
| Yellow Lemon | USA | Yellow | Saggitate, spotted | Pot-/Cut-flower |
| Royal Snowland | Netherlands | White | Lanceolate, spotted | Pot-flower |
| Medallion | USA | Orange | Saggitate, spotted | Pot-/Cut-flower |
| Santa Fe | Netherlands | Pink | Ovate, not spotted | Pot-flower |
| Cantor | Netherlands | Purple | Ovate, spotted | Pot-/Cut-flower |
