# Supplementary material for: Develop a preliminary core germplasm with the novel polymorphism EST-SSRs derived from three transcriptomes of colored calla lily (Zantedeschia hybrida)
Source: Front Plant Sci. 2023 Feb 2;14:1055881. doi: 10.3389/fpls.2023.1055881 (PMC9933510; doi:10.3389/fpls.2023.1055881)
Supplement: Supplementary Table 10 — The 42 core accessions of colored calla lily. [file Table_10.docx]

| **Number** | **Accessions** | **Country** | **Color** | **Leaves** | **Use Type** |
| --- | --- | --- | --- | --- | --- |
| A2 | Hong Yu | USA | Purple | Ovate, not spotted | Pot-flower |
| A6 | Neroli | New Zealand | Orange | Hastate, spotted | Pot-/Cut-flower |
| A10 | 8# | Unkown | Orange | Saggitate, spotted | Pot-/Cut-flower |
| A19 | B | Unkown | Pink | Ovate, spotted | Pot-flower |
| A21 | Black Magic | New Zealand | Yellow | Saggitate, spotted | Cut-flower |
| A24 | Wang A | New Zealand | Pink | Ovate, spotted | Pot-flower |
| A27 | Solid Gold | New Zealand | Yellow | Saggitate, spotted | Cut-flower |
| A30 | Mango | New Zealand | Orange | Hastate, spotted | Pot-/Cut-flower |
| A37 | 1# | Unkown | Yellow | Saggitate, spotted | Cut-flower |
| A40 | 6# | Unkown | White | Hastate, spotted | Cut-flower |
| A45 | Pacific Pink | New Zealand | Pink | Hastate, spotted | Cut-flower |
| A47 | Swan lake | New Zealand | White | Saggitate, spotted | Pot-/Cut-flower |
| A48 | Picasso | New Zealand | Purple | Saggitate, spotted | Pot-/Cut-flower |
| A49 | Aurora | New Zealand | Pink | Lanceolate, spotted | Pot-/Cut-flower |
| A51 | Romeo | New Zealand | Purple | Lanceolate, spotted | Pot-/Cut-flower |
| A53 | Rose Gem | USA | Pink | Lanceolate, not spotted | Pot-flower |
| A58 | Parfait | USA | Pink | Ovate, spotted | Pot-/Cut-flower |
| A60 | Flame | USA | Orange | Saggitate, spotted | Pot-/Cut-flower |
| A61 | Pillow Talk | USA | Pink | Lanceolate, not spotted | Pot-/Cut-flower |
| A64 | Super Gem | USA | Pink | Lanceolate, not spotted | Pot-flower |
| A68 | Allure | Netherlands | Purple | Ovate, spotted | Pot-flower |
| A73 | Aguila | Netherlands | Yellow | Saggitate, spotted | Pot-/Cut-flower |
| A81 | Maori | Netherlands | Purple | Saggitate, spotted | Pot-/Cut-flower |
| A85 | Paris | Netherlands | Purple | Hastate, spotted | Pot-/Cut-flower |
| A96 | Vermeer | Netherlands | Purple | Saggitate, spotted | Pot-/Cut-flower |
| A106 | Elmaro | New Zealand | Orange | Saggitate, spotted | Pot-/Cut-flower |
| A107 | Butter Gold | New Zealand | Yellow | Saggitate, spotted | Pot-/Cut-flower |
| A110 | Ice dancer | USA | White | Hastate, spotted | Pot-/Cut-flower |
| A116 | Yellow Lemon | USA | Yellow | Saggitate, spotted | Pot-/Cut-flower |
| A117 | Wanmei H | USA | White | Ovate, spotted | Pot-flower |
| A118 | Lemon Drop | New Zealand | Yellow | Saggitate, spotted | Pot-/Cut-flower |
| A121 | Mint Julip | USA | White | Lanceolate, not spotted | Pot-/Cut-flower |
| A123 | Neon Amour | USA | Pink | Lanceolate, not spotted | Pot-/Cut-flower |
| A132 | Royal Snowland | Netherlands | White | Lanceolate, spotted | Pot-flower |
| A142 | Medallion | USA | Orange | Saggitate, spotted | Pot-/Cut-flower |
| A144 | Lolly Pop | USA | Pink | Ovate, spotted | Pot-flower |
| A145 | Hot Flash | USA | Pink | Lanceolate, not spotted | Pot-flower |
| A153 | Santa Fe | Netherlands | Pink | Ovate, not spotted | Pot-flower |
| A154 | 41XW | Netherlands | Purple | Ovate, not spotted | Pot-flower |
| A155 | Belcanto | Netherlands | Purple | Ovate, not spotted | Pot-flower |
| A158 | Cantor | Netherlands | Purple | Ovate, spotted | Pot-/Cut-flower |
| A160 | Memphis | Netherlands | Yellow | Ovate, spotted | Pot-/Cut-flower |
